# Academic Competence, Teacher–Student Relationship, and Violence and Victimisation in Adolescents: The Classroom Climate as a Mediator

**DOI:** 10.3390/ijerph18031163

**Published:** 2021-01-28

**Authors:** Teresa I. Jiménez, David Moreno-Ruiz, Estefanía Estévez, Juan Evaristo Callejas-Jerónimo, Ginesa López-Crespo, Sonsoles Valdivia-Salas

**Affiliations:** 1 Department of Psychology and Sociology, University of Zaragoza, 44003 Teruel, Spain; tijimgut@unizar.es (T.I.J.); glopezcr@unizar.es (G.L.-C.); sonsoval@unizar.es (S.V.-S.); 2 Department of Social Psychology, University of Valencia, 46010 Valencia, Spain; 3 Department of Health Psychology, Miguel Hernández University, 03202 Alicante, Spain; eestevez@umh.es; 4 Department of Applied Economics I, University of Sevilla, 41018 Sevilla, Spain; jcallejas@us.es

**Keywords:** school violence, school victimisation, classroom climate, academic competence, teacher–student relationship, adolescents, mediation

## Abstract

School violence is a serious social and public health problem prevalent worldwide. Although the relevance of teacher and classroom factors is well established in the literature, few studies have focused on the role of teacher perceptions in school violence and victimisation and the potential mediational role of classroom climate in this relationship. A total of 2399 adolescents (50% girls), aged between 11 and 18 years (M = 14.65, SD = 1.78) and enrolled in five Spanish Secondary Compulsory Education schools completed measures of classroom climate, school violence towards peers and perception of peer victimisation, and their teachers informed about their academic competence and the teacher–student relationship. Correlational analyses revealed that whereas academic competence perceived by the teacher was negatively related to overt violence and victimisation, its relationship with pure relational violence was positive. Structural equation modelling analyses showed that variables of classroom climate (involvement, affiliation, and teacher support) perceived by the students functioned as partial mediators between teacher perceptions of academic competence and of teacher–student relationship and violence and victimisation. In the mediational model, teacher perception of academic competence acted as a direct protective factor against violence and victimisation, and teacher perception of teacher–student relationship acted as a direct risk for violence, as well as an indirect protective factor through classroom climate for victimisation. The interpretation of these results points to the importance of the teacher’s subjective perceptions in the prevention of violence and victimisation problems and their practical implications for the classroom climate perceived by students.

## 1. Introduction

School violence is a serious social and public health problem prevalent worldwide [1,2,3,4,5]. This problem greatly worries parents [6], teachers [7], researchers [8], and clinicians [9] due to the significant and negative—sometimes devastating—consequences for the physical and psychological health of the students involved [10,11,12], with long-term repercussions in the person’s integral development [13,14]. Among the different acts comprised in the term of school violence (i.e., aggression towards school staff, property damage, vandalism on the campus, bullying), the current study focused on violence perpetration towards peers, that is, on nonaccidental behaviours aimed at other classmates to cause harm intentionally; and on peer victimisation, that is, the perception of being exposed to negative actions by one or more students with the intention of hurting the victim [15]. These acts can manifest as physical (pushing, hitting, kicking, shoving, etc.), verbal (name-calling, taunting, threatening, etc.) or relational violence (spreading false rumours or malicious gossip, exclusion from activities, or withdrawal of friendships). The first two forms of violence–victimisation have been labelled as “direct or overt” bullying, and the third as “indirect or relational” bullying. To understand the relevant negative consequences of these behaviours (for a review see [16]), it is important to note that the perpetrator usually acts with the desire and intention to dominate and to exert control over the other person [17], creating a dynamic of violence from which the victims normally cannot defend themselves or escape by their own means, due to the imbalance of power between the people involved [18,19]. 

Considering prevalence, international data indicate great variation among the studies carried out in different countries. For example, in a study conducted with adolescents from 43 countries (Europe, USA, and Canada) by the World Health Organization (WHO) [20], the rates of bullying perpetration varied from 1 to 36%, and the rates of bullying victimisation between 2 and 32%. Hymel and Swearer [21] argued that the variation rate depends on the country of origin of the sample and the way that school violence was assessed. In adolescent Spanish samples, García-García et al. [22] conducted a systematic review of the prevalence of school violence in 32 studies, which yielded a rate of 11.45% for overall bullying. Moreover, in cross-cultural surveys, rates of violence perpetration and victimisation are consistently higher for boys than for girls [23]. More specifically, whereas initial research found that boys perpetrate and experience more overt violence, and that indirect forms of violence may be more typical of girls [24,25], later evidence suggests that both sexes are involved in similar levels of relational violence and victimisation [26,27].

In the search of causes for school violence and victimisation, many authors have explored the personal profiles of aggressors and victims and the characteristics of their family, school, and community environments (for a review from an ecological perspective, see [28]). Specifically, within the school context, most studies have focused on the student’s point of view by analyzing the perception of low teacher and peer support [12,29,30] or the importance of group processes such as the search for a social reputation and peer acceptance [28,31]. However, few studies have focused on the role that teachers’ perceptions of their students can play in the explanation of school violence and victimisation. Specifically, in this study, we focus on academic competence and the teacher–student relationship perceived by the teacher.

Concerning academic competence, previous studies in the field have shown a small but significant negative correlation between peer victimisation and academic achievement across studies (for a meta-analytic review, see [32]). In addition, a meta-analysis performed by Savage et al. [33] informs of the consistency of a negative and moderate relationship between academic achievement and violent antisocial behaviour. More specifically, traditional studies on bullying victimisation [15] have viewed aggression towards peers as a reaction to frustrations and failures at school. However, later studies provided no evidence for this hypothesis. For example, Woods and Wolke [34] found no relationship between academic achievement and direct forms of violence perpetration, and a positive and significant relationship with indirect or relational violence perpetration. However, most of these studies used academic achievement (i.e., grade point averages) as an objective indicator of academic competence and few of them have incorporated other teacher ratings of academic functioning, such as subjective ratings of effort and interest in classroom activities [32]. In this study, we are interested in extending previous literature in this field, considering a broader definition of academic competence because it has been shown that teachers’ subjective expectations about the performance of their students affect their interactions with them, influencing the classroom climate as a whole [35]. Moreover, it is well established that the quality of school and classroom climate is related to general antisocial behaviour [36] and, specifically, to violence and victimisation problems [11,12].

Concerning the teacher student–relationship, Bouchard and Smith [37] have recently argued that researchers and educators should recognize more fully the role of teacher–student relationship quality in students’ bullying–victimisation experiences. Drawing from the social–ecological system theory, these authors claimed the need to consider mesosystem analyses—such us teacher–student relationship quality—to deeply understand school violence and victimisation experiences. Specifically, some authors have argued that peer interactions in classrooms, including violence and victimisation, are implicitly shaped by the quality of teacher–student relationships because teacher–student interactions can be seen as a relational model [37,38,39]. Indeed, previous empirical evidence attests to the importance of teacher–student relationships in psychosocial development (for a review, see [40]). For example, students who report better relationships with teachers show higher self-esteem and prosocial behaviour, and fewer depressive and problem behaviours such as peer aggression [41,42]. Conversely, other studies have shown that teacher–student relationships that are distant and conflictive contribute to higher levels of school violence and victimisation [43,44]. Research has also revealed that low perceived support from teachers or perception of low teacher involvement is related to high school aggression [28,41,45]. However, most of the cited literature about the benefits of teacher–student relationships on psychosocial adjustment has focused on primary school and few studies have analysed these benefits in secondary schools, where the school structure does not allow teachers to have as many opportunities to develop significant and caring relationships with their students. Moreover, the existing literature has almost exclusively considered teacher–student relationships from the students’ point of view. This study aims to contribute to filling this gap by analysing the link between the teacher–student relationship perceived by the teacher and violence and victimisation in secondary school.

As noted before, teachers are one of the most influential agents in establishing the quality of classroom climate [46,47]. That is, teachers who engage in their practice and show affective attunement and support toward their students could contribute to generating a classroom climate where the students perceive support from others, participate in proposed activities, and view peers as friends [48]. Therefore, we can expect that the teacher’s factors have an indirect influence on the levels of violence and victimisation through their contribution to the classroom climate in which peers interact with each other. Indeed, some previous but scarce studies support the idea that classroom climate could serve as a mediator between teacher practices and peer violence [49]. Considering teacher perceptions, the original research conducted by Harris and Rosenthal [50] showed that teacher expectations about their students were related to academic outcomes through a classroom factor such as a warm and affective climate more than through the quality of dyadic interactions. Subsequent studies [35,51] have shown that teachers with high expectations for their students created a different instructional and socioemotional environment, characterized by a better structuring of the teaching process, more feedback, a greater number of open questions and more positive and preventive management of disruptive behaviour in the classroom. However, most of the previous literature focused on teacher perceptions and classroom climate have focused on academic outcomes and not on behavioural outcomes such as school violence and victimisation.

In sum, considering that (1) teacher perceptions of academic competence and of teacher–student relationship are related to school violence and victimisation, (2) teacher perceptions about their students affect classroom climate, and (3) classroom climate is related to violence and victimisation among peers, we might hypothesize that classroom climate could be a mechanism linking teacher perceptions and violence and victimisation. We have not found any previous studies in the field jointly analysing these factors, still less focusing on the teachers’ point of view.

### The Present Study

In accordance with the previous literature, in the present study, we seek to (1) explore the relationships between the study variables (i.e., academic competence and teacher–student relationship perceived by teachers, classroom climate, school violence, and school victimisation); (2) determine whether classroom climate mediates the relationship between teacher perceptions of academic competence and of teacher–student relationship, and school violence and victimisation; and (3) examine the role of sex as a potential moderator of these relationships in a sample of Spanish adolescents. We expect to find the mediating role of classroom climate and no sex differences in the pattern of mediational relationships among the study variables.

## 2. Materials and Methods

### 2.1. Procedure

The data we are analysing in the present study were collected as part of a larger study on violent behaviour in Spanish adolescents. Initially, a letter with a summary of the research project was sent to the principal of each school to explain the purpose of the research and to request permission to carry out the study. The management team of each school requested the collaboration of their teacher staff. Then, we requested parents’ and guardians’ consent for the students to participate in this study (only 1% did not give consent). We explained the relevance and the goals of the study to the students and informed them that participation was voluntary and anonymous. The administration of the instruments was carried out by a group of trained researchers with at least one qualified researcher (with a Ph.D.) present during the administration of the instruments to provide students with the necessary support. Measures were collected in the classrooms during a regular class period without the presence of the teachers. In parallel, the homeroom teachers (the teacher who is in charge of taking care of students’ academic and personal-related affairs in a specific classroom during an academic course) filled in the PROF-A scale. The research was conducted in compliance with ethical standards required for research with human beings, respecting the basic principles included in the Helsinki Declaration and approved by the Ethics Committee of the Vice-Chancellor for Research of the host university. 

### 2.2. Participants 

The study was an ex post facto cross-sectional and descriptive study. Participants in the study were 2399 adolescents (50% girls), aged between 11 and 18 years (*M* = 14.65, *SD* = 1.78), enrolled in five state schools of Secondary Compulsory Education and High School located in the Valencian Community (Spain). Sample distribution by academic grade, family composition, and parents’ educational level is presented in Table 1.

### 2.3. Instruments

#### 2.3.1. Academic Competence and Teacher–Student Relationship

To evaluate academic competence and teacher–student relationship, we used two of the subscales of the School Adjustment Perceived by the Teacher Scale (Escala de Ajuste Escolar Percibido por el Profesor—PROF-A) developed by Cava et al. [52]. This scale was designed to assess the teachers’ perception of different factors related to their students’ social adjustment and academic performance by using 14 items. Academic competence contains four items (e.g., “Student’s participation in activities, discussions, debates, etc., proposed in class”) and the teacher–student relationship subscale includes four items (e.g., “The time I spend talking to this student”). Items are rated on a 10-point scale ranging from 1 (very low) to 10 (very high). Adequate psychometric properties were obtained in previous studies performed with samples of secondary students [52,53], with Cronbach’s alpha values ranging from 0.85 to 0.92. In our study, the Cronbach’s alpha obtained for academic competence was 0.94 and 0.85 for the teacher–student relationship. In the confirmatory factorial analysis (CFA), the chi-square values and fit indexes were *χ*^2^(103) = 1137.31, *p* < 0.001, RMSEA (root mean square error of approximation) = 0.06 [0.06, 0.07], and CFI (comparative fit index) = 0.96.

#### 2.3.2. Classroom Environment

We used the relationship dimension of the classroom environment scale [54] in the Spanish version of Fernández-Ballesteros and Sierra [55]. This scale evaluates classroom environment from the point of view of the students through 27 binary-choice (true–false) items, forming three subscales: (1) Involvement (degree of student attentiveness, interest, and participation in class activities; 9 items, e.g., ‘‘Students put a lot of energy into what they do here’’); (2) Affiliation (degree of friendship and support among students; 9 items, e.g., ‘‘Students in this class get to know each other really well’’); and (3) Teacher support (amount of help, trust, and friendship the teacher offers to students; 9 items, e.g., ‘‘The teacher takes a personal interest in the students”). The Cronbach’s alpha values obtained in previous studies with Spanish adolescent samples ranged between 0.77 and 0.89 (e.g., [41,56]). In our sample, Cronbach’s alpha of the global scale was 0.77 and 0.66, and 0.64 and 0.63 for the three subscales, respectively. In the CFA analysis, the chi-square values and fit indexes were *χ*^2^(368) = 1075.66, *p* < 0.001, RMSEA = 0.03 [0.03, 0.04], and CFI = 0.91.

#### 2.3.3. School Violence

This scale by Little et al. [57] (bidirectional translation into Spanish, using the parallel back-translation procedure of Brislin [58]) includes 25 items that assess participation in aggressive behaviour towards peers at school over the last 12 months. It is rated on a 4-point Likert-type scale ranging from 1 (never) to 4 (always). The scale evaluates six dimensions of violence within two types of aggressive behaviour: overt or direct and relational or indirect; and three functions of violence: pure, reactive, and instrumental. These dimensions are pure overt (e.g., “I’m the kind of person who hits, kicks, or punches others”); reactive overt (e.g., “If others make me upset or hurt me, I often put them down”); instrumental overt (e.g., “I often threaten others to get what I want”); pure relational (e.g., “I’m the kind of person who says mean things about others”); reactive relational (e.g., “If others have threatened me, I often say mean things about them”); and instrumental relational (e.g., “To get what I want, I often ignore or stop talking to others”). In the present study, all items were summed to generate six composite variables that fed the school violence construct in the measurement model. The Cronbach’s alpha values obtained in previous studies with Spanish adolescent samples ranged between 0.64 and 0.87 [12,56,59]. In our sample, Cronbach’s alpha of the global scale using these composites was 0.85 (the reliability range for the six subscales varied from 0.63 in instrumental relational to 0.77 in reactive overt). In the CFA analysis, the chi-square values and fit indexes were *χ*^2^(178) = 641.02, *p* < 0.001, RMSEA = 0.03 [0.03, 0.04], and CFI = 0.91.

#### 2.3.4. School Victimisation

The peer–victimisation scale developed by Cava et al. [60] is a measure of self-reported victimisation based on the multidimensional peer–victimisation scale of Mynard and Joseph [61] and the social experience questionnaire self-report of Crick and Grotpeter [25]. This scale consists of 20 items that assess the frequency with which the students had experienced 20 victimizing experiences in the last school year on a response range of 1 (never) to 4 (always). The scale presents a three-factor structure: relational victimisation (e.g., “a peer got angry with me and separated me from my group of friends to prevent me from playing or participating in any activity”); overt physical victimisation (e.g., “a peer hit me to really harm me”); and overt verbal victimisation (e.g., “a peer insulted me”). The psychometric properties of the scale are adequate in previous studies [60,62] and in the present study, with internal consistencies (Cronbach α) of the three subscales ranging from 0.68 to 0.92 (0.92 in the present study for the total scale and 0.89, 0.63, and 0.84 for the three subscales, respectively). In the CFA analysis, the chi-square value and fit indexes were *χ*^2^(159) = 664.28, *p* < 0.001, RMSEA = 0.04 [0.03, 0.04], and CFI = 0.93.

### 2.4. Statistical Analysis

Firstly, descriptive statistics (means and standard deviations) and Pearson’s correlations were computed using SPSS (Version 25.0, IBM, Armonk, NY, USA). Secondly, multivariate inferential analyses were conducted using structural equation modelling (SEM) through Mplus 8.4 software [63]. Complementary analyses were also performed to determine the significance and magnitude of the potential mediating effect [64,65]. Finally, to further check the robustness of the proposed model, we tested it using structural invariance across sex through multigroup analyses.

The maximum likelihood estimate with robust standard errors (MLR) was used to correct the non-normality of the data [63]. The fixed models were evaluated using a combination of indices, including the comparative fit index set and the Tucker–Lewis index (CFI/TLI > 0.90 for a reasonable fit and CFI/TLI > 0.95 for a good fit), the mean square root of the residuals (SRMR < 0.09 for a good fit), and the mean square root of the approximation (RMSEA < 0.06 for a proper fit) [66]. Although a chi-square test (S-B χ^2^) for model fit was also reported, it was not used to evaluate model fit due to its sensitivity to large sample sizes. Throughout the whole study, the reported path coefficients were standardised values. To perform the test of the indirect effect, its confidence intervals were calculated using the bootstrap method with 2000 samples.

## 3. Results

### 3.1. Descriptive Analysis

Regarding the descriptive statistics and correlations for the observed variables, Table 2 shows the means, standard deviations, and bivariate correlations. In general, variables of school adjustment perceived by the teacher (academic competence and teacher–student relationship) were positively and significantly related to variables of classroom climate (involvement, affiliation, and teacher support perceived by the students), with *r* ranging from 0.063 to 0.116, *p* < 0.01. Academic competence and variables of school violence and victimisation were negatively and significantly related (*r* ranging from −0.040, *p* < 0.05, for instrumental relational violence to −0.239, *p* < 0.01, for reactive overt violence) except for pure relational violence (this bivariate correlation was positive with *r* = 0.045, *p* < 0.05). Teacher–student relationship was negatively and significantly related to overt reactive violence (*r* = −0.085, *p* < 0.01) and overt physical victimisation (*r* = −0.047, *p* < 0.05), but this relationship was positive with pure violence (both overt and relational; *r* = 0.043, *p* < 0.05 and *r* = 0.077, *p* < 0.01, respectively). All bivariate correlations between classroom climate and violence and victimisation variables were negative and significant (*r* ranging from −0.051, *p* < 0.05, to −0.232, *p* < 0.01).

### 3.2. Structural Equation Modelling

To analyse the direct relationship between school adjustment and school violence and victimisation, and indirectly through classroom climate, a structural equation model (SEM) was created. Table 3 shows the latent variables included in the models, their respective indicators, the standard error, and the associated probability for each indicator in the corresponding latent variable.

**Figure 1:**
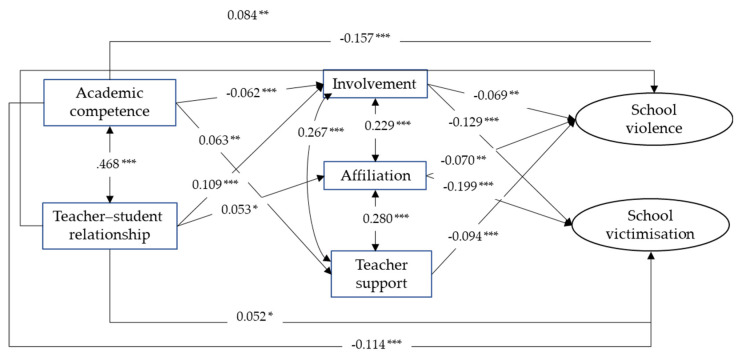
Final structural model with path coefficients and statistical significance. *** *p* < 0.001; ** *p* < 0.01; * *p* < 0.05.

The calculated model (see Figure 1) showed an adequate fit to data: S-B χ^2^ = 461.304, *df* = 45, *p* < 0.001, CFI = 0.952, RMSEA = 0.062 [0.057, 0.067]. A direct and negative relationship was observed between teacher perception of academic competence and school violence (β = −0.157, *p* < 0.001), and between teacher perception of academic competence and school victimisation (β = −0.114, *p* < 0.001). The direct relationship was positive, although smaller, between teacher perception of teacher–student relationship and school violence (β = 0.084, *p* < 0.01) and victimisation (β = 0.052, *p* < 0.05). Moreover, the three classroom climate variables (involvement, affiliation, and teacher support) were directly and negatively related to school violence (β = −0.069, *p* < 0.01; β = −0.070, *p* < 0.01; β = −0.094, *p* < 0.001), and two of them (involvement and affiliation) to school victimisation (β = −0.129 and β = −0.199, *p* < 0.001). The percentage of variability of school violence and victimisation explained by the structural model was 15.1% (9.6% for victimisation and 5.5% for violence), which can be considered to be a size of the effect of the statistical significance of the estimated model.

Regarding the indirect relationships or mediational effects, we calculated all of them and the results showed multiple significant indirect effects of academic competence and teacher–student relationship on school violence and victimisation through the three variables of classroom climate. Their magnitude ranged from −0.001 (e.g., Academic competence → Involvement → Teacher support → School violence) to −0.014 (Teacher–student relationship → Involvement → School victimisation); that is, all of them were very small. Total indirect effects, direct effects, and total effects are presented in Table 4. Overall, the total effect of teacher perception of academic competence on school violence and victimization was negative (i.e., protective, higher academic competence was associated to lower violence and victimization) and higher than total effect of teacher perception of teacher–student relationship (in this case total effects were positive, i.e., higher teacher–student relationship was associated to higher violence and victimization). Concerning the effect of teacher perception of academic competence, both direct and indirect effects were negative and, in consequence, the total effects were higher and significant (β = −0.164 for violence and β = −0.122 for victimization, *p* < 0.001). Regarding the effect of teacher perception of teacher–student relationship both, direct and indirect effects, were smaller and opposite (a risk if direct and a protection if indirect) and, in consequence, total effects were lower and only significant, as a risk, for school violence (β = 0.067, *p* < 0.05).

Finally, to check the robustness of this model, we tested its structural invariance across sex groups (boys and girls) through a multigroup analysis. Two models were tested: in the unrestricted model, parameter estimates (factor loadings and structural paths) were freely estimated across groups; in the restricted model, we constrained each of the factor loadings and the structural paths to be invariant across groups. If the chi-square of the restricted model was significantly larger than the chi-square of the unrestricted model, the assumption of invariance would not be tenable. The restricted model showed a fit of S-B χ^2^ = 939.430, *df* = 139, *p* < 0.001, CFI = 0.907, RMSEA = 0.69 [0.065, 0.074]. The results showed significant differences between boys and girls in the model (∆χ^2^_(35, 2399)_ = 103.432, *p* < 0.001). Observing the analyses and using index modification, three paths presented significant differences between the sex groups: first, the relationship between teacher support and school violence was negative and significant in girls (β = −0.148, *p* < 0.001) but higher in boys (β = −0.165, *p* < 0.001); second, the loading of instrumental overt violence in school violence was positive and significant in girls (β = −0.448, *p* < 0.001) but higher in boys (β = −0.585, *p* < 0.001); and finally, the relationship between affiliation and school violence was negative and significant in girls (β = −0.098, *p* < 0.001) but higher in boys (β = −0.112, *p* < 0.001). Once the restrictions were released, both models were shown to be equivalent for boys and girls (Δχ^2^_(30, 2399)_ = 32.735, *p* > 0.05).

## 4. Discussion

The main objectives of the present study were to explore the relationships between teacher perceptions of academic competence and of teacher–student relationship, students’ perception of classroom climate, and violence and victimisation in a sample of Spanish secondary students, and to determine whether classroom climate mediates the relationship between teacher perceptions and school violence and victimisation. We expected to find evidence for the mediating role of classroom climate and no sex differences in the pattern of relationships among the study variables. Overall, our findings yielded support for the proposed mediational model, showing evidence for a partial mediation of classroom for both sexes with the consideration of small sex differences in some paths. Specifically, our results point to the idea that teacher perception of academic competence acts as a direct protection against school violence and victimization; however, relational violence towards peers seems not to be a reaction to academic failures at school because it related to a better academic competence perceived by the teacher. In addition, we observe that the teacher’s perception of a good teacher–student relationship could act as a direct risk factor for school violence and an indirect protective factor for school victimisation through the improvement of the classroom climate perceived by the students.

Regarding our first objective, all correlations between variables of teacher perceptions (academic competence and teacher–student relationship) and variables of classroom climate (involvement, affiliation, and teacher support) were significant and positive. That is, greater levels of academic competence and greater teacher–student relationships evaluated by the teacher were related to better classroom climate evaluated by the students (involvement in activities, perception of friendship and support among classmates, and perceived teacher support). These results are coherent with previous studies [35,67], adding evidence in favour of the positive role of teachers’ perceptions about their students for creating a positive and supportive climate for the students. In addition, as expected and in coherence with previous literature (e.g., [28]), all classroom climate variables (involvement, affiliation between classmates, and teacher support) were significantly related to lower levels of school violence and victimisation. We have also found evidence of a differential link between teacher perception of academic competence and school violence as a function of the form of violence used to harm peers; in our results, academic competence perceived by the teacher was negatively related to overt or direct violence but positively related to pure relational or indirect violence. As some previous studies have pointed out, it seems that relationally violent adolescents in school settings are successful students [34,68]. This result reinforces the idea that violence towards peers is not a reaction to frustrations and failures at school, as initially argued by Olweus [15], and suggests the need to pay attention to the fact that relational violence could be more invisible to adults because it is exerted by “good” students. Finally, it is important to note that we found a positive relationship between the teacher perception of teacher–student relationship and pure violence (both overt and relational). In other words, students receiving a higher evaluation from their teachers in terms of the relationship with them showed more violence towards peers (both pure overt and pure relational). This unexpected result will be discussed later.

In line with our second objective, we found evidence for the mediational role of classroom climate between teacher perceptions and school violence and victimisation. This mediational effect was partial (only indirect teacher–student relationship effects were significant) with significant direct and indirect, although small, effects. The fact that the mediation was partial means that, in spite of the mediational role of classroom climate in the tested model (indirect effects), there is a considerable amount of variance in school violence and victimisation that is directly related to the teacher perception of academic competence and of teacher–student relationship (direct effects). Next, we will first discuss academic competence results and then we will examine the teacher–student relationship results.

Regarding the direct effect of academic competence, we found evidence for a protective effect against violence as well as against victimisation. In addition, indirect effects through the mediation of classroom climate were negative, although nonsignificant, and total effects confirmed the protective role of academic competence against the involvement in violence towards peers and the perception of being victimised. On the whole, this is in line with the literature that has consistently related academic achievement and violence and victimisation [32,33,34]. Our results add to previous evidence on the link between academic achievement and bullying victimisation, so that, in addition to results with more objective indicators of academic achievement (grade point averages or reported grades), we now know that this protective link is confirmed with subjective indicators of academic performance (academic competence perceived by the teacher). Research suggests that some teachers incorporate behaviours, such as effort and participation, into final classroom grades [69]. Thus, we consider it more accurate to explicitly incorporate these subjective evaluations into the academic indicator, even more so when previous literature in the field of teacher expectations have confirmed the relevance of teachers’ subjective expectations on school academic outcomes [35,70,71]. Our results extend previous literature linking teacher perceptions of academic competence to school behavioural outcomes such as violence and victimisation.

Considering results related with the teacher perception of teacher–student relationship, we found evidence for a direct but smaller risk effect for violence and victimisation, and for a protective effect when classroom climate acts as a mediator. On the whole, the total effect of the teacher–student relationship on school violence remained positive and significant after considering indirect effects, but it was nonsignificant in the case of victimisation. Some considerations are needed to deeply understand these results. The measure of teacher–student relationship used in this study includes four items in which the teacher assesses his/her relationship with the student, the student’s relationship with other teachers, the teacher’s personal interest shown to the student, and the time the teacher spends talking with the student. Regarding direct effects, it seems that a more intensive relationship with a student could be a risk factor for being violent towards peers and also being victimised. Although these results seem controversial, they are very interesting to understand the important role that the teacher plays in the processes of peer violence and victimisation at school. On the one hand, the teacher’s personal interest shown to some aggressive students could lead to a reinforcement of the problematic behaviour [72] (Alberto and Troutman, 2012). In this line, in a recent longitudinal study, it was observed that teacher support perceived by the student was a risk for long-term aggression (at the beginning of the next course) when the teacher’s perceived support was maintained throughout the course [30]. On the other hand, the teacher’s personal interest shown to some students who have characteristics related to a high risk of victimisation (e.g., low self-esteem, loneliness, social anxiety) [11,73] could lead to a reinforcement of the victim role in the eyes of their peers. In line with this idea, in a longitudinal study, it was observed that dependency on the teacher (assessed by the teacher) predicted heightened victimisation from peers, and that decreases in the number of friendships mediated the link between dependency on the teacher and heightened relational victimisation for boys [74]. To prove these hypothetical explanations, it would be necessary to include some personal student variables in the tested model within longitudinal studies and, even more so to complement studies with specific naturalist observations to analyse in detail student–teacher interactions throughout a course.

In parallel, our results also show a protective effect of the teacher perception of teacher–student relationship through the mediation of an increment in a student’s perceived classroom climate. In other words, a better relationship with the student informed by the teacher is related to a better student’s perception of the classroom climate (perceptions of more involvement, friendship, and support among classmates, and perceived teacher support) and this is, in turn, related to lower levels of violence and victimisation. This result is coherent with previous literature where teachers have been regarded as one of the most influential agents in establishing the quality of classroom climate [46,47]. It is important to note that the total indirect effect of the teacher–student relationship on victimisation was almost twice the amount of indirect effect of the teacher–student relationship on violence. Thus, in the case of victimisation, the indirect protective effect of the teacher–student relationship compensated for the direct risk effect. This entails a greater indirect benefit of the teacher–student relationship on victimisation (a good relationship with a student could lead the student to feel better in the classroom and less victimised) than on violence. This dual role of the teacher–student relationship (as a risk and as protection) in school violence and victimisation could be paradoxical, and more research is needed to clarify it. However, we consider that these results add evidence to previous socioecological literature [37] for understanding more deeply the relevant influence of mesosystem variables such as teacher–student relationships on adolescents’ social development and for identifying interpersonal factors associated with involvement in peer harassment.

Finally, multigroup analyses showed that the proposed mediational model was equivalent for both sexes after controlling for some sex differences. First, the relationship between teacher support perceived by the student and school violence was negative for both sexes, but higher in boys. This difference was significant but small, and we can interpret it as possible sex differences in the preferred way of exercising violence. Traditional and recent studies have consistently found that boys are more involved in peer violence than girls, especially in its overt or direct form (for a review, see [26]), that is, in a kind of violence more “visible” to adults. Then, it is possible that, when boys perceive support from their teachers, they feel more connected to them and, perhaps, more observed by them, and, in consequence, feel more restrained in their behaviour. Second, the relationship between affiliation (perception of friendship and support among students) and school violence was negative for both sexes but also higher in boys. Some previous studies have shown that girls tend to perceive more relational aggression from their friends than do boys [75]. Then, classroom friendship might be perceived as a more secure and protective context for boys than for girls. Overall, further research is necessary to clarify these observed sex differences.

### Limitations and Future Research

Considering that we have been dealing with sensitive information that could be subjected to social desirability (i.e., violence and victimisation), one limitation of the present study is the use of self-administered anonymous questionnaires as the only source of information for dependent variables. Although some findings have indicated that using only self-reports could be efficient for examining the relationship between bullying behaviours and some child characteristics [76], complementing the study with peer reports might lead to a broader and more comprehensive approach to this topic. In addition, the percentage of variability of school violence and victimisation explained by the mediational model tested was low (15.1%). This means that the proposed model contributes to the understanding of school violence and victimisation, but alternative models considering other mesosystem variables (e.g., parent–teacher relationships), microsystem (e.g., peer relationships), and youth characteristics (e.g., empathy) are needed to reach a broader picture of the violence–victimisation problem at school [28,37]. Even more so, as mediation was partial, this means that other variables could explain the mechanism through which teacher perceptions of academic competence and of teacher–student relationships are related to school violence and victimisation. Additional research is called for to complete this mediational mechanism. Moreover, the use of cross-sectional data poses a statistical limitation, as it is well known that cross-sectional studies are not the best to analyse mediation [77]. Cross-sectional tests of mediation may yield statistical bias that could be solved with longitudinal data sets. Therefore, to confirm the relations observed in the present study, longitudinal mediational analysis should be conducted. Finally, it is worth noting that the study revealed significant associations of very small effect sizes, which may reflect not only causal relationships, but the effect of a relatively large sample size as well. Therefore, other studies that could replicate these findings are needed.

## 5. Conclusions

In conclusion, despite these and other possible limitations, we highlight the contribution of our findings to the field of school violence and victimisation. Our results provide a better understanding of the empirical relationship between teachers’ perceptions of academic competence and of teacher–student relationship, and violence and victimisation problems at school. Teacher perception of academic competence acted as a direct protective factor against violence and victimisation, and teacher perception of teacher–student relationship acted as a direct risk for violence, as well as an indirect protective factor through classroom climate for victimisation. Hence, some aspects can be outlined regarding the scope of violence and victimisation prevention. First, any intervention in schools would need to focus on teachers’ perceptions of their students’ academic competence. Teachers having a positive and broad concept of the academic competence of their students, not only focused on their average grades, could be protecting them from becoming involved in violence and victimisation. Second, it is necessary to promote teachers as healthy relational models for their students, developing a high sensitivity to properly manage the type of attention and relational intensity needed in the relationship with each student.

## Figures and Tables

**Table 1:** Sociodemographic characteristics of the sample.

Sociodemographic Characteristics	%
Academic Grade	
1st grade secondary	21.4
2nd grade secondary	20.1
3rd grade secondary	18.2
4th grade secondary	18.6
1st grade high school	10.2
2nd grade high school	11.5
Family Composition	
Intact	83.5
Single parent	15.7
Living with other adults	0.8
Mother Educational Level	
Higher education	35.9
High school	16.4
Primary school	25.8
No studies	8
Unknown	13.9
Father Educational Level	
Higher education	37.8
High school	14.7
Primary school	22
No studies	8.4
Unknown	17.1

**Table 2:** Pearson’s correlations and descriptive statistics for observed variables.

	1	2	3	4	5	6	7	8	9	10	11	12	13	14
1. Academic competence	-													
2. Teacher–student relationship	0.468 **	-												
3. Involvement	0.028	0.116 **	-											
4. Affiliation	0.079 **	0.086 **	0.310 **	-										
5. Teacher support	0.078 **	0.063 **	0.334 **	0.286 **	-									
6. Pure overt violence	−0.067 **	0.043 *	−0.097 **	−0.105 **	−0.149 **	-								
7. Reactive overt violence	−0.239 **	−0.085 **	−0.125 **	−0.102 **	−0.118 **	0.460 **	-							
8. Instrumental overt violence	−0.090 **	0.012	−0.067 **	−0.119 **	−0.127 **	0.504 **	0.398 **	-						
9. Pure relational violence	0.045 *	0.077 **	−0.051 *	−0.086 **	−0.119 **	0.417 **	0.205 **	0.412 **	-					
10. Reactive relational violence	0.021	0.030	−0.110 **	−0.083 **	−0.119 **	0.288 **	0.309 **	0.302 **	0.438 **	-				
11. Instrumental relational violence	−0.040 *	0.004	−0.072 **	−0.106 **	−0.097 **	0.327 **	0.257 **	0.603 **	0.456 **	0.401 **	-			
12. Relational victimisation	−0.066**	−0.003	−0.173 **	−0.232 **	−0.125 **	0.139 **	0.039	0.160 **	0.176 **	0.164 **	0.186 **	-		
13. Overt physical victimisation	−0.123**	−0.047 *	−0.108 **	−0.160 **	−0.093 **	0.144 **	0.146 **	0.139 **	0.091 **	0.017	0.092 **	0.496 **	-	
14. Overt verbal victimisation	−0.086**	−0.039	−0.197 **	−0.222 **	−0.148 **	0.203 **	0.117 **	0.174 **	0.155 **	0.117 **	0.148 **	0.752 **	0.604 **	-
Mean	5.927	7.388	1.455	1.717	1.597	1.366	1.578	1.133	1.332	1.778	1.192	1.471	1.180	1.593
Standard deviation	2.028	1.313	0.208	0.173	0.220	0.322	0.542	0.243	0.323	0.470	0.305	0.439	0.284	0.470

Note: ** *p* < 0.01; * *p* < 0.05.

**Table 3:** Factorial loadings, standard error, and associated probability.

Factors and Variables	Factor Loadings (Standard Errors)
School Violence	
Pure overt violence	1 ^a^
Reactive overt violence	1.834 *** (0.432)
Instrumental overt violence	0.656 *** (0.073)
Pure relational violence	0.750 *** (0.081)
Reactive relational violence	0.861 *** (0.101)
Instrumental relational violence	0.811 *** (0.087)
School Victimisation	
Relational victimisation	1 ^a^
Overt physical victimisation	0.883 *** (0.068)
Overt verbal victimisation	0.958 *** (0.068)

Note: Robust statistics. Standard errors in brackets. ^a^ Fixed at 1 during estimation. *** *p* < 0.001.

**Table 4:** Indirect, direct, and total effects with their confidence intervals.

	**β**	**Standard** **Error (s)**	**95% CI**		***p***
			**CL**	**UL**	
**Total Indirect Effects**					
Academic competence → School violence	−0.007	0.005	−0.015	0.001	0.005
Teacher–student relationship → School violence	−0.017 **	0.005	−0.025	−0.008	0.005
Academic competence → School victimisation	−0.008	0.007	−0.020	0.003	0.007
Teacher–student relationship → School victimisation	−0.031 ***	0.007	−0.042	−0.020	0.007
**Direct Effects**					
Academic competence → School violence	−0.157 ***	0.031	−0.208	−0.106	0.031
Teacher–student relationship → School violence	0.084 **	0.028	0.038	0.129	0.028
Academic competence → School victimisation	−0.114 ***	0.025	−0.155	−0.072	0.025
Teacher–student relationship → School victimisation	0.052 *	0.025	0.012	0.093	0.025
**Total Effects**					
Academic competence → School violence	−0.164 ***	0.031	−0.215	−0.114	0.031
Teacher–student relationship → School violence	0.067 *	0.027	0.023	0.111	0.027
Academic competence → School victimisation	−0.122 ***	0.026	−0.165	−0.079	0.026
Teacher–student relationship → School victimisation	0.021	0.026	−0.021	0.064	0.026
	**β**	**Standard** **Error (s)**	***p***	**95% CI**	
				**LCL**	**UCL**
**Total Indirect Effects**					
Academic competence → School violence	−0.007	0.005	0.149	−0.015	0.001
Teacher–student relationship → School violence	−0.017 **	0.005	0.001	−0.025	−0.008
Academic competence → School victimisation	−0.008	0.007	0.237	−0.020	0.003
Teacher–student relationship → School victimisation	−0.031 ***	0.007	0.000	−0.042	−0.020
**Direct Effects**					
Academic competence → School violence	−0.157 ***	0.031	0.000	−0.208	−0.106
Teacher–student relationship → School violence	0.084 **	0.028	0.002	0.038	0.129
Academic competence → School victimisation	−0.114 ***	0.025	0.000	−0.155	−0.072
Teacher–student relationship → School victimisation	0.052 *	0.025	0.035	0.012	0.093
**Total Effects**					
Academic competence → School violence	−0.164 ***	0.031	0.000	−0.215	−0.114
Teacher–student relationship → School violence	0.067 *	0.027	0.012	0.023	0.111
Academic competence → School victimisation	−0.122 ***	0.026	0.000	−0.165	−0.079
Teacher–student relationship → School victimisation	0.021	0.026	0.406	−0.021	0.064

Note: CI = confidence interval; LL = lower limit; UL = upper limit; *** *p* < 0.001; ** *p* < 0.01; * *p* < 0.05.
